# Brain Plasticity and Disease: A Matter of Inhibition

**DOI:** 10.1155/2011/286073

**Published:** 2011-07-03

**Authors:** Laura Baroncelli, Chiara Braschi, Maria Spolidoro, Tatjana Begenisic, Lamberto Maffei, Alessandro Sale

**Affiliations:** ^1^National Research Council (CNR), Neuroscience Institute, Via Moruzzi 1, I-56124 Pisa, Italy; ^2^Department of Psychology, Florence University, Pizza San Marco 4, I-50121 Florence, Italy; ^3^Laboratory of Neurobiology, Scuola Normale Superiore, Pizza Cavalieri 7, I-56126 Pisa, Italy

## Abstract

One major goal in Neuroscience is the development of strategies promoting neural plasticity in the adult central nervous system, when functional recovery from brain disease and injury is limited. New evidence has underscored a pivotal role for cortical inhibitory circuitries in regulating plasticity both during development and in adulthood. This paper summarizes recent findings showing that the inhibition-excitation balance controls adult brain plasticity and is at the core of the pathogenesis of neurodevelopmental disorders like autism, Down syndrome, and Rett syndrome.

## 1. Introduction

The term “plasticity” refers to the ability of the nervous system to reorganize its connections functionally and structurally in response to changes in environmental experience, underlying the adaptive development of neuronal circuitry. The existence of time windows in early postnatal life (critical periods) during which neural circuits display a heightened plasticity in response to external stimuli has been established for various brain regions subserving major behavioural functions (for review, see [1, 2]). After the end of the critical period, neural plasticity dramatically wanes. Since the pioneering work by Wiesel and Hubel, the visual system stands as the prime model for studying experience-dependent plasticity. These authors reported that occluding one eye early in development (a treatment usually referred to as monocular deprivation) leads to an ocular dominance shift of cortical neurons, that is, a reduction in the number of cortical cells responding to that eye and a robust increment in the number of neurons activated by the open eye [3]. The imbalance of activity between the two eyes eventually results in the loss of synaptic inputs from the thalamic regions representing the closed eye and in the expansion of those driven by the open eye [4–7], accompanied by a remodelling of cortical horizontal connections [8]. 

In the last 50 years, great effort has been made to elucidate cellular and molecular mechanisms underlying the activation and regulation of critical periods in the brain. Unravelling these processes may potentially enable researchers to enhance plasticity in the adult brain. Moreover, a detailed knowledge of the events involved in the maturation and plasticity of neuronal circuitry would be a determinant in improving our understanding of the aetiology of developmental brain disorders. 

Although a complete picture in the field is still lacking, a large body of evidence has been accumulated (see, [9, 10]). In this paper, we will focus our discussion on intracortical inhibitory circuitry which convincingly emerges as a key factor not only for defining the boundaries of cortical plasticity but also in developing of pathological states characterized by severe intellectual disabilities (see also [11, 12]).

## 2. GABAergic Inhibition and Ocular Dominance Plasticity in the Adult Visual Cortex

By sculpting the pattern and timing of neuronal electrical activity, inhibitory GABAergic circuits are an ideal candidate for regulating the processes of experience-dependent synaptic modifications. Taking advantage of gene-targeting technology, this hypothesis has been directly tested by abolishing the expression of the 65 kD isoform of GABA-synthetic enzyme, hence reducing activity-dependent GABA synthesis and release at synaptic terminals. Mice that carry such a disruption of the GAD65 gene do not exhibit ocular dominance plasticity in response to monocular deprivation; only an enhancement of inhibition achieved with local delivery of diazepam enables a full rescue of ocular dominance plasticity in these mice [13]. 

Converging results obtained with different experimental approaches have subsequently confirmed the key role of GABAergic inhibition in brain development and plasticity (e.g., [14–17]). It is noteworthy that BDNF-overexpressing mice show an accelerated maturation of GABAergic cortical inhibition paralleled by a faster time course of critical period for ocular dominance plasticity [13], strongly suggesting that the progressive development of the inhibitory tone not only enables the onset of the critical period but subsequently underlies the closure of neural plasticity gates.

One of the major challenges in neuroscience is the development of strategies aimed at promoting nervous system plasticity in adulthood, when recovery from injury and functional rehabilitation are severely hampered. Recently, new evidence has challenged the classic dogma that ocular dominance plasticity is a physiological phenomenon exclusively restricted to the early postnatal development and pointed to a reduction of intracortical inhibition levels as a crucial step for the restoration of plasticity processes in the adult brain. The most direct demonstration that GABAergic inhibition limits plasticity in the adult visual cortex derives from a recent study reporting that pharmacological reduction of intracortical inhibition obtained through the infusion of either MPA (an inhibitor of GABA synthesis) or picrotoxin (a GABA_A_ antagonist) directly into the visual cortex reactivates ocular dominance plasticity in response to monocular deprivation in adult rats [18]. Moreover, this treatment leads to a full rescue of long-term potentiation (LTP) of layer II-III field potentials induced by theta-burst stimulation from the white matter, an activity-dependent form of synaptic plasticity which is normally occluded in visual cortical slices from adult animals due to the maturation of inhibitory transmission [18, 19]. The reduction of intracortical inhibition is accompanied by processes of structural plasticity. The visual cortex of MPA or PTX-treated animals, indeed, shows a decrease in the density of chondroitin sulfate proteoglycans (CSPGs), indicating the activation of endogenous mechanisms of extracellular matrix remodelling which are known to be crucially involved in the expression of neural plasticity [20–22]. It is also possible that other molecular components of the extracellular milieu regulating synaptic plasticity in the adult brain, such as myelin proteins [23] and adhesion molecules [24], may undergo changes in their expression levels in response to a reduction of intracortical inhibition.

These results show that a brief reduction of GABAergic inhibition is sufficient to reopen a window of plasticity in the visual cortex well after the normal closure of the critical period. Similar conclusions have been drawn from recent evidence showing that the inhibitory tone is a central hub for the restoration of plasticity in the adult visual cortex and that a decrease of intracortical inhibition levels is required for the reinstatement of neural plasticity triggered by different experimental approaches. We demonstrated that environmental enrichment, a condition of increased sensory-motor and cognitive stimulation, reactivates juvenile-like ocular dominance plasticity in the visual cortex of adult rats, with a shift in ocular dominance of cortical neurons following monocular deprivation clearly detectable using both visual evoked potentials and single-unit recordings [25]. Recovery of plasticity in enriched animals is paralleled by a marked reduction of the inhibitory tone in the visual cortex. Importantly, the decrease of inhibitory neurotransmission is a crucial molecular mechanism underlying the enhancement of visual cortex plasticity induced by environmental enrichment: preventing the reduction of GABAergic inhibition during the period of exposure to environmental enrichment (via micro-osmotic pumps infusing the GABA agonist diazepam into the visual cortex), indeed, completely blocks the ocular dominance shift of cortical neurons in response to monocular deprivation [25]. The enhanced environmental stimulation provided by environmental enrichment also leads to a twofold enhancement of serotoninergic transmission and to an increase in the number of BDNF-expressing neurons in the visual cortex. Interestingly, infusion of a serotonin synthesis inhibitor not only blocks plasticity in response to monocular deprivation but also fully counteracts the effects produced by environmental enrichment on inhibition and BDNF levels. We suggested a model in which serotonin is the first trigger in the molecular chain set in motion by environmental enrichment, eliciting the decrease of GABA-mediated intracortical inhibition and, in parallel or in series, the enhancement of BDNF levels [25]. 

It is interesting to point out that while, during development, environmental enrichment increases BDNF and accelerates the maturation of inhibition in the visual cortex [15], in adult animals reared in an enriched environment increased levels of BDNF are associated with reduced GABAergic inhibition. One possible explanation for these apparently contrasting results is that the influence exerted by the environment on these molecular factors may follow a temporarily distinct sequence in the adult compared to the developing brain. Specifically, we propose that the very early (postnatal day 7, see [15]) increase in BDNF detected in mice reared from birth in an enriched environment may be the prime factor that directly drives the development of inhibitory circuitry in the immature brain; on the contrary, the enhancement of BDNF expression in animals exposed to environmental enrichment in adulthood may occur downstream to the decrease of intracortical inhibition, which could promote the expression of many activity-dependent genes involved in neural plasticity. 

Given the central role of serotonin in promoting adult visual cortex plasticity, one might expect that the effects induced by environmental enrichment should be reproducible through an artificial modulation of cerebral levels of this neurotransmitter. This possibility has been addressed in a study by Maya Vetencourt et al. [26], showing that the administration of fluoxetine, a selective serotonin reuptake inhibitor (SSRI) widely prescribed in the treatment of depression for its capability to enhance extracellular serotonin levels, reinstates plasticity in the visual cortex of adult animals, with treated rats exhibiting a marked shift of ocular dominance in favour of the open eye after one week of monocular deprivation. Also in this case, a pronounced reduction of intracortical inhibition has been detected in the visual cortex, and the osmotic infusion of the GABA agonist diazepam fully prevents the ocular dominance shift induced by monocular deprivation, thus impeding plasticity in fluoxetine-treated animals. Further support to the notion that diffuse projecting systems of the brainstem affect plasticity in adulthood has been very recently provided by the demonstration that a genetic enhancement of nicotinic cholinergic transmission restores ocular dominance plasticity well after the end of the critical period. This effect is abolished by diazepam treatment, suggesting that the cholinergic signalling mechanisms may adjust excitatory-inhibitory balance [27]. 

Using an approach quite different from environmental enrichment, a study by He and colleagues reported that exposing adult animals to complete darkness can also promote plasticity in the visual cortex [28]. These authors provided indirect evidence that the enhanced cortical plasticity might be related to a shift in the balance between inhibition and excitation towards levels more similar to those found in the immature cortex, caused by a reduced expression of GABA_A_ receptors relative to AMPA receptors. This suggestion has been confirmed in a very recent study [29] showing that dark exposure decreases inhibitory synaptic density and paired-pulse depression and reinstates in the visual cortex the expression of endocannabinoid-dependent inhibitory long-term depression, a form of synaptic plasticity normally restricted to the juvenile age [30]. 

Two different hypotheses, not mutually exclusive, could be formulated for explaining how the reduction of the inhibitory tone to juvenile-like levels leads to a recovery of cerebral plasticity in the adult brain. According to one hypothesis, the maturation of GABAergic intracortical transmission sets the point after which the editing activity of visual cortex pyramidal neurons enables ocular dominance plasticity; as development proceeds further, the inhibitory tone surpasses a threshold, and this causes the closure of the critical period. A reduction of inhibition levels may reinstate in the adult visual cortex the capability of binocular neurons to detect the imbalance in retinal inputs induced by the closure of one eye. According to an alternative hypothesis, the overall increase of cortical activity due to the shift in excitation-inhibition balance is the key factor favoring plasticity recovery. Activity-dependent regulation of gene expression could induce a genetic transcriptional program critical for promoting plasticity.

## 3. Beyond the Visual Cortex

The critical role of GABAergic inhibition in regulating experience-dependent plasticity is not restricted to the visual cortex. 

In the barn owl, the optic tectum contains a map of space consisting of bimodal neurons whose auditory and visual receptive fields are mutually aligned. In juvenile owls, alternative maps of interaural time difference can be acquired as a result of abnormal experience. The group of Knudsen and colleagues has demonstrated the existence of a sensitive period for plasticity in the optic tectum by exposing owls at different ages to prismatic spectacles that cause a large horizontal shift of the visual field [31]. Owls bearing these spectacles experience a modification of the visual locations to which the interaural time difference values correspond, eliciting the adjustment of auditory receptive fields according to the optical displacement [31, 32]. Very interestingly, the environmental rearing conditions can have a dramatic impact on this form of plasticity. Indeed, the period during which owls respond adaptively to prismatic displacement of the visual field ends at about 70 days of age when owls are housed in individual cages, while it does not end until 200 days of age when owls are housed in groups and in larger enriched rooms [31]. At the same manner, also the ability to recover after restoration of normal visual experience is strongly affected by the environment, because it ends at 200 days of age when prism-reared owls are housed in small cages but extends throughout life when they are housed in group flight rooms. Soon after the characterization of the sensitive period for visual calibration of the auditory space map, Zheng and Knudsen demonstrated that when a new learned map is expressed in the external nucleus of the owl optic tectum, the neural circuitry underlying the old map is not structurally inactivated but becomes silent due to a functional suppression operated by inhibitory connections and involving GABAA receptors [33]. 

In the mammalian auditory system, a well-defined critical period exists for tone-specific enlargement in the primary auditory cortex (A1) representation resulting from transient exposure to sound stimuli [34]. Strikingly, the Merzenich's group has recently demonstrated that while in adult control rats this exposure produces no measurable alteration of A1 tonotopy, rats transferred to an environment of continuous moderate-level noise exhibit a re-establishment of a period of sound exposure-driven plasticity [35]. This effect, which is reminiscent of the reopening of critical period plasticity triggered in the visual system by dark exposure, is paralleled by a decrease in the expression level of GABAA *α*1 and *β*2/3 subunits in A1. 

Thus, reduction of GABAergic inhibition may emerge as a common feature of the strategies that successfully reopen a period of stimulus exposure-based plasticity in the adult brain [18, 25, 26, 28, 35].

## 4. Pathological Inhibition of Cerebral Function: The Case of Amblyopia

During the critical period, the high susceptibility of neuronal connections to experience-dependent changes is essential for a proper maturation of normal sensory functions. This high potential for plasticity, however, may also favour the emergence of developmental pathological states when an anomalous perturbation of sensory-driven activity takes place. A paradigmatic case is that of amblyopia, a widely diffused and still untreatable pathology of the visual system affecting 2–4% of the total world population [36]. Amblyopia derives from conditions of early abnormal visual experience in which a functional imbalance between the two eyes is predominant owing to anisometropia (unequal refractive power in the two eyes), strabismus (abnormal alignment of one or both eyes), or congenital cataract, resulting in a dramatic loss of visual acuity and a broad range of other perceptual abnormalities, including deficits in stereopsis and contrast sensitivity [37, 38]. It is worth stressing that in amblyopic patients the visual impairment is caused by an abnormal processing of visual information at the central level; thus, the use of corrective lenses is completely ineffective [39–41]. 

It is currently accepted that, due to a lack of sufficient residual plasticity within the brain, the reinstatement of visual functions in amblyopic subjects is possible only if corrective treatment is started early in development. The classic amblyopia therapy consists in patching or penalizing the preferred eye, thus forcing the brain to use the visual input carried by the weaker amblyopic eye [42]. However, an increasing number of clinical and animal studies are now challenging these traditional beliefs, reporting that repetitive visual training based on sensory enrichment procedures may represent a very useful approach for the treatment of amblyopia (for a comprehensive review, see [38, 43]). 

The mechanisms underlying vision improvements in adult amblyopic patients remain to be elucidated, since the activation of cortical plasticity may occur at several different levels of the visual system and through a variety of neural processes. A number of studies, however, suggested that an impairment of the balance between excitation and inhibition could affect visual cortex development and that cortical overinhibition could underlie the degradation of spatial vision abilities [44–48]. Accordingly, recent advances in our understanding of the cellular and molecular brakes that limit amblyopia recovery to a critical period underscored intracortical inhibition as a main obstacle for reinstatement of normal visual functions after a period of early abnormal visual experience. In animal models, amblyopia can be induced by imposing a long-term reduction of inputs from one eye by lid suture (i.e., with a protocol of long-term monocular deprivation). Similarly to that observed in humans, animals rendered amblyopic by long-term monocular deprivation display a permanent loss of visual acuity in the affected eye and a pronounced ocular dominance shift of visual cortical neurons in favour of the normal eye (e.g., [49–51]). 

Early studies in animal models of amblyopia reported that the administration of anti-inhibitory compounds (e.g., bicuculline) leads to a substantial restoration of binocularity in the visual cortex [52, 53]. Recently, it has been shown that the same experimental paradigms discussed in Section 2 and associated with a reduced inhibition-excitation balance in the adult cerebral cortex are also able to recover sight from amblyopia (for review [54, 55]). Among these treatments, environmental enrichment emerges as a totally non-invasive approach [56]. We reported that a brief exposure (two-three weeks) of adult amblyopic rats to environmental enrichment promotes a complete recovery of both visual acuity and ocular dominance, as demonstrated both with electrophysiological recordings of visual evoked potentials from the primary visual cortex and with a standard visual acuity behavioural test (visual water-box task). The environmental enrichment-induced recovery of visual acuity is long-lasting, persisting for a minimum of two weeks [56]. A reduced intracortical inhibition is a crucial mechanism underlying the enhancement of visual cortex plasticity in environmental enrichment: preventing the reduction of GABAergic inhibition during the period of environmental enrichment, indeed, completely blocks the recovery of binocularity and visual acuity. These findings draw attention to the environmental enrichment procedure as a prospective, injury-free, intervention strategy for amblyopia and further substantiate a major role for GABAergic transmission in the control of plasticity windows in the sensory cortices.

## 5. Inhibition and Neurodevelopmental Disorders

While the physiological maturation of GABAergic connections is essential for a tight control of developmental cortical plasticity and for promoting the acquisition of mature sensory abilities, it is currently accepted that abnormal levels of inhibition achieved during development can cause pathological states of severe brain disability [11, 57, 58]. On this regard, Rett syndrome, Down syndrome, and autism disorder stand as the most informative cases (the role of inhibition in schizophrenia is discussed in another review published in this issue). 

### 5.1. Rett Syndrome

Rett syndrome is a progressive developmental disorder characterised by mental retardation and severe dysfunction in motor coordination skills [59], predominantly affecting the female population in early childhood. Using a systematic gene screening approach, loss-of-function mutations in the X-linked gene encoding the methyl-CpG binding protein (MeCP2) have been identified as the cause of Rett syndrome [60]. MeCP2 is involved in the regulation of expression of a wide range of genes [61] and in RNA splicing [62]. Transgenic mice carrying conditional deletion or neuron specific expression of mutated MeCP2 forms exhibit abnormalities in motor coordination, social interaction, and cognitive abilities, providing a useful model for analysing the behavioural and molecular phenotype of the Rett syndrome [63–66]. 

Detailed electrophysiological analysis of these animal models showed a reduction of neuronal activity in cortical and hippocampal neurons due to a shift in the balance between cortical excitation and inhibition in favour of inhibition [67, 68] and an attenuation of LTP expression in the hippocampus and in the motor and somatosensory cortex [68, 69]. These results led to the hypothesis that an anomalous increase in the inhibition/excitation ratio could be responsible for the motor, behavioural, and cognitive defects associated with Rett syndrome [11]. This interpretation is supported by autoradiographic labelling studies on human postmortem brain samples, showing a significant increase in the density of GABA receptors that may correlates with cognitive and motor symptoms of Rett syndrome [70]. A very recent work by Chao and colleagues further demonstrated that a dysregulation of GABAergic system has a role in modulating the pathogenesis of Rett syndrome: mice lacking MeCP2 selectively in GABA-releasing neurons, indeed, recapitulate most of the behavioural features of Rett syndrome [71]. Surprisingly, these mice display a reduced inhibitory tone, while no data were presented concerning levels of excitation. Therefore, while these results confirm that a dysfunction of GABAergic neurons can contribute to the Rett phenotype, they also outline a more complex framework for the involvement of inhibitory transmission in Rett syndrome. 

Since the gene encoding BDNF is under MeCP2 regulation [72] and the severity of behavioural symptoms in MeCP2 deficient mice correlate with levels of circulating BDNF [73], attempts have been made to rescue the Rett syndrome phenotype by delivering BDNF. It has been shown that exogenous BDNF in MeCP2 mutant mice is able to compensate for deficits at the behavioural, anatomical, and electrophysiological level [73, 74]. Pre-weaning environmental enrichment, which results in augmented cerebral BDNF levels, ameliorates motor and cognitive impairment and reverses cortical LTP deficits [75]. Very interestingly, environmental enrichment increases the number of cortical excitatory synapses with no changes found in inhibitory synaptic density, thus resulting in overall reduction of the cortical inhibitory tone [75].

### 5.2. Down Syndrome

Down syndrome is caused by tri-plication of chromosome 21 (Chr21) and is the most common genetic cause of mental retardation [76]. People with Down syndrome have moderate to severe cognitive impairment, with various disturbances in learning and memory abilities [77, 78]. In search of possible molecular and cellular processes involved in the pathogenesis of the syndrome, several murine models have been generated, carrying triplications of different segments of Chr16, which has a large degree of synteny with human Chr21 [79, 80]. Currently, the prime model is the Ts65Dn transgenic mouse [81, 82], which recapitulates all main hallmarks of the Down syndrome phenotype, including characteristic craniofacial abnormalities, impaired spatial and nonspatial learning abilities, and attention and visual function deficits (e.g., [83–85]). Anatomical studies indicated that Ts65Dn mice have a reduced number of cerebellar and hippocampal neurons [86–88], impaired neurogenesis in the dentate gyrus of the hippocampus (see [86, 89]; see also [90] for similar evidence in human foetuses), and simplified dendritic branching in several brain regions, associated with alterations in spine size and shape [91–93]. Moreover, dysfunctions in the mechanisms driving nerve-growth factor (NGF) retrograde transport from the hippocampus to the basal forebrain [94, 95] are responsible for a prominent degeneration of basal forebrain cholinergic neurons in adult Ts65Dn mice [96], which is also a hallmark of the Alzheimer's disease. Accordingly, nearly one hundred per cent of persons born with Down syndrome develop Alzheimer's disease if they live into their fourth decade of life [96, 97]. 

A large number of studies have shown that the cognitive impairment displayed by Ts65Dn mice is mainly related to excessive levels of inhibition in temporal lobe circuitry, causing a failure of long-term synaptic plasticity in the hippocampus [98–100]. The deficit of synaptic plasticity is linked to marked morphological changes in the structure of synapses, with a selective enlargement of the active zones of symmetric synapses and increased immunostaining for synaptic proteins localized at inhibitory synapses in cortex and hippocampus [101, 102]. The central role of overinhibition in Down syndrome pathogenesis has been recently confirmed by the demonstration that administration of non-competitive antagonists of GABAA receptors reverses spatial learning disabilities and LTP deficits in Ts65Dn mice [100]. 

One of the major challenging tasks in the field of Down syndrome therapy is unravelling dosage-sensitive genes whose dysfunction, due to the presence of an extra copy of chromosome 21, might be responsible for the main functional and morphological defects. A recent study by Chakrabarti et al. [103] has shown that two genes, Olig1 and Olig2, are essentially involved in the syndrome. The authors first reported that, very early in development, Ts65Dn mice have a marked increase in the number of forebrain GABAergic neurons generated in the medial ganglionic eminence (one of two regions in the ventral telencephalon where most inhibitory neurons proliferate and differentiate). More specifically, an overproduction of two specific classes of inhibitory neurons (i.e., parvalbumin- and somatostatin-positive neurons) has been detected. This anatomical phenotype is directly related to increased levels of inhibitory transmission in the forebrain of Ts65Dn mice, as assessed with electrophysiological methods [103]. Remarkably, a genetic reinstatement of dysomia at the level of Olig1 and Olig2 genes (obtained by breeding Ts65Dn mice with a line having only one copy of each of these genes) was sufficient to rescue the Ts65Dn phenotype, correcting the interneuron overproduction and restoring synaptic transmission to euploid levels [103]. Even if a behavioural assessment of the cognitive performance in Ts65Dn mice after re-establishment of dysomia was not reported, these results suggest that a few dosage-sensitive genes might eventually be responsible for many of the deficits displayed by people with Down syndrome and further support a causal link between aberrant inhibition in cortical and hippocampal circuitries and cognitive impairment due to Down syndrome. 

Despite the increasing knowledge concerning the molecular mechanisms underlying Down syndrome, a suitable treatment for this disorder is still lacking. Since environmental enrichment is particularly effective in reducing GABAergic inhibition [104], it may have a great potential for therapeutic application to Down syndrome. Martínez-Cué et al. have reported increased exploratory behaviour and enhanced spatial learning in enriched Ts65Dn mice, albeit the effect was gender-specific [105]. Despite these results, a detailed investigation of the environmental enrichment effects on Down syndrome pathogenesis is still lacking.

### 5.3. Autism

Autism is a heterogeneous developmental disorder characterised by significant impairments in the social, communicative, and cognitive domain and by the presence of repetitive patterns of stereotyped activities [106, 107], mostly affecting males in early childhood [108]. The advent of magnetic resonance imaging enabled the *in vivo* investigation of structural brain morphology in people with autism. Several regions have been reported to be enlarged or reduced relative to controls, but a large consensus on these results is currently missing (for a review, [109]).

The aetiological mechanisms of autism are at present poorly defined. Despite a likely contribution of environmental causes, genes play a crucial role in the onset of this pathology with concordance between monozygotic twins reaching 90%, as compared with less than 10% for dizygotic twins and siblings [110, 111]. Only recently, considerable efforts have been focused on understanding the genetic basis of autism and led to the identification of multiple chromosomal loci and epigenetic factors associated with autism heritability (for a review, [112]). Given the complex repertoire of symptoms characterising autistic syndrome, it has been proposed that defects in the development and functioning of multiple and relatively independent neural systems work together to generate the pathological phenotype. In particular, neural circuits underlying social and emotional behaviour, language processing, and higher-order cognition are considered natural candidates [113]. 

Converging results have pointed to an increased excitation/inhibition ratio in sensory, mnemonic, social, and emotional systems as a core mechanism underlying neurological and behavioural deficits observed in autistic patients [58]. Consistently, clinical studies showed that epilepsy displays a good percentage of comorbidity with autism [114]. An imbalance of neural circuits leading to a disproportionate high level of excitation could be due to increased glutamatergic transmission or suppressed GABAergic inhibition. The hypothesis that a reduction of inhibitory neurotransmission shared in common between many systems could be a key factor in the pathogenesis of autism is consistent with a large body of evidence [115]. Indeed, a significant reduction in protein levels of both isoforms of glutamic acid decarboxylase [116, 117] and GABA receptors [118–120] has been reported in autistic cerebral cortex. Linkage genetic studies uncovered that polymorphism, copy number, and epigenetic alterations in chromosomal regions containing GABA receptor subunit genes are associated with autistic phenotype [121–123]. 

On the cellular level, it has been shown that in a valproic acid rat model of autism, the amygdala is hyperreactive to electrical stimulation and displays enhanced synaptic plasticity as well as defective inhibitory transmission [124]. Moreover, a direct demonstration that inhibitory circuitries are activated atypically and are less synchronized in the brain of autistic people has been provided by studies of functional magnetic resonance imaging [125, 126]. 

Since autism is a developmental disorder, the imbalance in the ratio of excitation versus inhibition could result from abnormal processes during neural circuit maturation. Indeed, defects in synaptogenesis and synaptic refinement have been suggested to be a leading cause of autism, and mutations of genes that normally control the patterning of synaptic maturation of specific neuronal subpopulations have been shown to segregate with the pathological phenotype [127, 128]. Among these genes, Dlx1 and Dlx2 encode transcription factors exerting a crucial role in the generation of GABAergic cortical interneurons and lie in a chromosomal region associated with autism susceptibility [129]. In accordance with the excitation/inhibition model, it has been proposed that pharmacological agents that reduce neural excitation, such as anticonvulsivants and benzodiazepines, could represent a suitable therapeutic treatment for autism [58]. At present, some evidence that anticonvulsivants could be effective in ameliorating autistic symptoms is available (e.g., [130–132]). 

It should be pointed out, however, that the exact role of excitation/inhibition balance in autism is still debated. Indeed, an increased inhibitory synaptic transmission and a decreased glutamatergic excitation have been also reported in different transgenic mouse models of autism [133, 134].

## 6. Concluding Remarks

Altogether the results reviewed here show how dramatic can be the influence exerted by inhibitory transmission on brain plasticity. Not only are these findings crucial to our knowledge about the molecular mechanisms underlying the expression and regulation of plasticity processes, but they also have strong implications for the treatment of neurological disorders related to an aberrant development of GABAergic circuits. The possibility of rescuing a normal phenotype in animal models of these pathologies by manipulating levels of intracortical inhibition draws attention on the GABAergic system as an eligible candidate for the development of new therapeutic strategies.
